# OX40/OX40L: a new target for tumor immunotherapy and its clinical research progress

**DOI:** 10.3389/fonc.2025.1730620

**Published:** 2026-01-05

**Authors:** Zixuan Yuan, Yutong Pan, Haodong Yuan, Aihua Gong

**Affiliations:** School of Medicine, Jiangsu University, Zhenjiang, Jiangsu, China

**Keywords:** OX40, OX40L, tumor immunity, targeted therapy, OX40 agonist

## Abstract

TNFRSF4 (OX40) -TNFSF4 (OX40L) axis is the core costimulatory pathway in the TNF/TNFR superfamily that regulates T cell responses. The binding of OX40L to OX40 on the surface of activated T cells significantly enhanced the proliferation and survival of CD4^+^ and CD8^+^ T cells and the secretion of IFN-γ and IL-2, while inhibiting the immunosuppressive activity of regulatory T cells, thereby amplifying the anti-tumor immunity. However, although OX40 agonist monotherapy is well tolerated in phase I/II clinical trials, the objective response rate is lower than that of PD-1 monotherapy, and it does not significantly prolong progression-free survival. At the mechanistic level, insufficient affinity, limited infiltration of T cells in the tumor and residual regulatory T cells are considered to be the main bottlenecks. Despite higher objective response rates with the addition of PD-1, radiotherapy, or chemotherapy, grade 3–4 immune-related adverse events were associated with higher rates. In the future, novel OX40 agonists with high affinity and selective activation in the tumor microenvironment should be developed or incorporated into the framework of combined immunotherapy as an adjuvant strategy to achieve a balance between efficacy and safety.

## 1 Introduction

In the field of oncology, immunotherapy has rapidly evolved into a paradigm-shifting therapeutic modality that potentiates host antitumor immunity to eradicate malignant cells within the tumor microenvironment (1). Although surgery, radiotherapy, and chemotherapy remain the cornerstones of oncologic management, their therapeutic efficacy is often restricted in advanced or relapsed/refractory malignancies and is accompanied by considerable toxicity. In recent years, immunotherapy has achieved precise and durable tumor cell elimination by reinvigorating host immunity, thereby redefining the therapeutic paradigm for numerous solid and hematologic neoplasms (2, 3). Among the immunotherapeutic strategies, the OX40/OX40L costimulatory axis is pivotal in initiating and amplifying immune responses and has become a focal point of translational research. Immune checkpoints encompass stimulatory or inhibitory signals mediated by membrane-bound receptor–ligand pairs, intracellular enzymes, or secreted molecules that preserve the dynamic equilibrium between pathogen clearance and prevention of autoimmunity (4, 5). Tumors exploit this regulatory circuitry by upregulating checkpoint molecules, resulting in immune escape (6, 7). Checkpoint inhibition, as illustrated by monoclonal antibodies directed against programmed cell death protein-1/programmed death-ligand 1 (PD-1/PD-L1) and cytotoxic T-lymphocyte antigen-4 (CTLA-4), has markedly extended overall survival among individuals with advanced-stage malignancies (8). Nevertheless, many tumors remain refractory owing to scarce T cell infiltration, underscoring the imperative for innovative strategies to reactivate antitumor immunity. OX40 signaling can attenuate Treg-mediated immunosuppression and reconfigure the functional patterns of B cells, dendritic cells, tumor-associated macrophages, and natural killer cells, thereby inhibiting tumor progression (9–12). By means of fusion protein design, nanoparticle-mediated plasmid delivery, and recombinant viral vectors, etc., genetic engineering techniques are used to control the expression of OX40L (13–15), investigators can substantially enhance the antitumor potency of both conventional T lymphocytes and chimeric antigen receptor–modified T (CAR-T) cells. Several OX40 agonist antibodies have been developed and have demonstrated promising activity in experimental animal systems and initial human studies (16). However, there is no unified standard for the optimal timing and dose of administration, and the best combination of treatments has not been found. Studies on the same dosing strategy in different patients are lacking. This results in highly variable dosing patterns in clinical trials, which are difficult to compare. In addition, immune-related adverse events (irAEs) have also occurred in different degrees during the treatment of OX40 agonists, and some agonist studies have been terminated due to insufficient efficacy, which is the main challenge in the development of OX40 agonists. This article reviews the OX40/OX40L pathway related molecular structure, signaling mechanism, regulatory role in tumor microenvironment and targeted therapy strategies. This review also summarizes some clinical trials using OX40 agonists, analyzes the problems and limitations of current treatment methods, proposes solutions, and proposes future directions.

## 2 Biological characteristics and functions of OX40/OX40L

OX40 is a type I transmembrane glycoprotein, part of the TNFR superfamily, with an estimated molecular weight of 48 to 50 kDa, located on chromosome 1 and clustered with other TNFR members at 1p36 (17). The initial identification of OX40 occurred on activated CD4+ T cells in mice and was subsequently detected in human cells (18). Current evidence indicates that OX40 is broadly expressed on activated CD4+ and CD8+ T cells and is detectable in dendritic cells (DC), natural killer (NK) cells, and epithelial cells (19, 20).

OX40L belongs to the TNF superfamily and functions as a type II transmembrane glycoprotein. It is tightly linked to TNFSF6 and is located on chromosome 1q25. OX40L is a 183 amino acids polypeptide with a molecular mass of approximately 34–40 kDa. It is mainly expressed in professional antigen-presenting cells and is present in tissues including the heart, bone, and lung. Activated T cells additionally express OX40L at low levels (21).

The OX40/OX40L axis promotes dendritic cell maturation and differentiation, enhances intercellular adhesion, amplifies T cell function, facilitates helper T cell (Th) polarization, and sustains T cell activity and persistence (13, 22). Professional antigen-presenting cells (APC) provide the primary signal via the major histocompatibility complex–peptide engagement with the T cell receptor (TCR) and the second signal through B7–CD28 interactions. Activated T cells upregulate OX40 and TNFSF5 (CD40L), which engages TNFRSF5 (CD40) on APC, increasing APC OX40L expression and modulating the axis. OX40/OX40L action proceeds sequentially: (a) in lymph nodes, guided by this signal, CD4+ T cells that have undergone activation relocate into B-cell follicles, where they facilitate germinal-center development and antibody secretion; (b) OX40-mediated co-stimulation licenses activated CD4+ T lymphocytes to egress into the circulation and subsequently home to sites of inflammation; (c) tissue-derived OX40L+ APC mediates local inflammatory effects of CD4+ T cells in peripheral tissues (23–26).

## 3 Overview of signaling pathways

**Figure 1:**
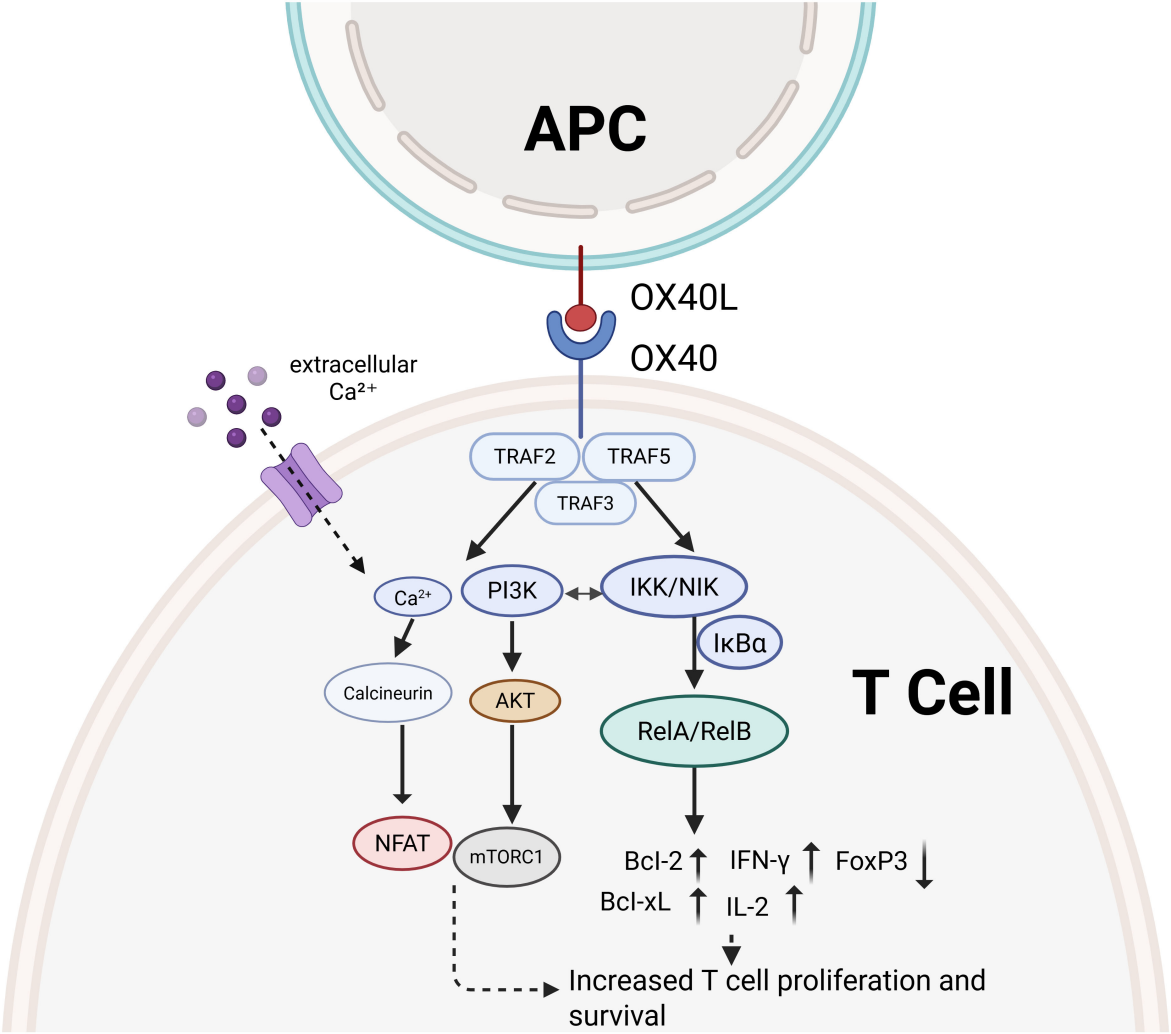
The interaction between OX40 and OX40L and its downstream signaling pathways can lead to T cell proliferation and survival. It can promote the secretion of cytokines and inhibit the transcription of FoxP3, thereby reducing tumor immunosuppression by suppressing regulatory T cells.

The core mechanism of OX40/OX40L signaling involves sequential kinase cascades and transcription factor activation, ultimately modulating T cells by regulating the expression of genes associated with cell survival, metabolism, and differentiation. **Figure 1** illustrate the OX40/OX40L interaction and its downstream signaling processes leading to improved T cell activation, proliferation, and survival. In addition, it can reduce the transcription of FOXP3 and inhibit Treg cells, thereby alleviating tumor immunosuppression.

### 3.1 PI3K/AKT pathway

Recruit tumor necrosis factor receptor-associated factor 2 and 5 (TRAF2/5) adaptor proteins following OX40 engagement, which initiates a signaling cascade. This process activates phosphatidylinositol 3-kinase (PI3K) and subsequently induces phosphorylation of protein kinase B (AKT). Activated AKT significantly enhances T cell survival and amplifies effector responses through the transcriptional repression of pro-apoptotic factors Bim and Bad, coupled with the upregulation of anti-apoptotic proteins Bcl-2 and Bcl-xL (27). Through its activation of mechanistic target of rapamycin complex 1, AKT promotes metabolic reprogramming and enhances the proliferation of antigen-specific T-cell clones (28).

### 3.2 The NF-κB pathway

OX40 signaling activates both the canonical and noncanonical nuclear factor kappa B (NF-κB) pathways through TRAF-dependent mechanisms. Within the canonical cascade, phosphorylation of NF-κB inhibitory protein α (IκBα) by the IκB kinase complex liberates NF-κB heterodimers, which then migrate into the nucleus to initiate the expression of proinflammatory mediators including interleukin-2 (IL-2) and interferon-γ (IFN-γ) (29). Activation of the RelB–p52 heterodimer in the noncanonical cascade depends upon NF-κB-inducing kinase, thereby contributing to memory T cell formation (30, 31).

### 3.3 MAPK pathway

Ligation of OX40 to OX40L triggers simultaneous activation of p38 mitogen-activated protein kinase (p38 MAPK) and c-Jun N-terminal kinase, leading to enhanced activator protein 1 transcriptional activity. p38 MAPK not only stabilizes mRNA encoding type 2 helper T cell (Th2) cytokines such as interleukin-4 and interleukin-5, but also phosphorylates MAPK-interacting kinase 1 to facilitate eukaryotic initiation factor 4E-mediated protein translation, thereby promoting the differentiation of both Th2 and type 17 helper T cell (Th17) subsets (24, 32).

### 3.4 Calcium ion signaling and PKC activation

Upon OX40 engagement, phospholipase C is activated and cleaves phosphatidylinositol-4,5-bisphosphate into inositol-1,4,5-trisphosphate and diacylglycerol. Inositol-1,4,5-trisphosphate triggers endoplasmic reticulum Ca²^+^ release, elevates intracellular free Ca²^+^ levels, activates calcineurin, and consequently drives the nuclear import of nuclear factor of activated T cells (33, 34). Diacylglycerol subsequently triggers protein kinase C (PKC), particularly PKCβ2, which enhances NF-κB signaling through phosphorylation of IκBα and facilitates T cell migration to inflammatory sites (35).

## 4 Role of OX40 pathway in tumor immunity

### 4.1 Enhanced T cell activity and persistence

OX40/OX40L exerts direct stimulatory effects on stimulated CD4^+^ and CD8^+^ T cells, orchestrating activation, multiplication and functional persistence via convergent signaling cascades (36, 37). Through canonical NF-κB and PI3K–Akt modules, the axis amplifies TCR-mediated activation while upregulating anti-apoptotic Bcl-2 family proteins, thereby preventing activation-induced cell death and prolonging T cell viability (38, 39). OX40L potentiates CD8^+^ cytotoxic T lymphocyte effector functions by inducing IL-2 and IFN-γ secretion, which augments tumor-antigen recognition and clonal burst size (40, 41). Furthermore, OX40L mitigates the suppressive effects imposed by PD-1/PD-L1, CTLA-4 and T cell immunoglobulin and mucin-domain containing-3 (TIM-3), thereby antagonizing these inhibitory checkpoints. It mitigates PD-1-driven exhaustion, attenuates Treg-mediated suppression and promotes secretion of granzyme B and IFN-γ, thereby potentiating cytotoxicity and restoring effector T cell competence (42–44). Positive feedback loops established via upregulated costimulatory receptors further lower the activation threshold (45). OX40L also programs transcriptional networks that promote the conversion of effector T cells into long-lived central memory T and stem-cell-like memory T subsets. These memory subsets demonstrate superior self-renewal, long-term persistence, rapid clonal expansion and robust effector cytokine production upon antigen re-encounter, thereby sustaining durable immune surveillance (46, 47). Experimental data demonstrate that concurrent OX40L agonism and PD-1 pathway inhibition significantly augments intratumoral memory T cell frequency and prolongs antitumor immunity (48).

### 4.2 Functional effects on regulatory T cells

Regulatory T cells play a central inhibitory role in the tumor microenvironment and inhibit the function of effector immune cells through a variety of mechanisms, thereby promoting tumor escape from immune surveillance and leading to tumor growth and metastasis (49, 50).Tregs directly inhibit the killing activity of CD8+ T cells by secreting inhibitory cytokines such as IL-10 and TGF-β, and hinder the migration and activation of T cells (51). Moreover, they maintain immune tolerance and inhibit the effective recognition and immune response to tumor-associated antigens by integrating microenvironmental signals such as tumor-associated antigens (52). When OX40 is expressed on Tregs and activated by OX40L, it impairs the suppressive function of Tregs by reducing the expression of the key transcription factor Foxp3, as well as inhibiting Treg differentiation and reducing their cellular activity, impairing the ability of Tregs to secrete inhibitory cytokines (53, 54). This mechanism is particularly obvious in the tumor microenvironment and can eliminate the immunosuppressive effect of Treg, thereby promoting anti-tumor immunity. Although Treg cells also express OX40, the role of OX40 signaling in Treg is two-sided. On the one hand, OX40 activation can transiently inhibit the immunosuppressive function of Treg and weaken its inhibitory effect on effector T cells. On the other hand, long-term OX40 signaling may promote Treg expansion (55, 56). In cancer therapy, OX40 agonists can attenuate Treg immunosuppression and enhance effector T cell activity (57). In chronic inflammatory diseases, OX40/OX40L axis has been regarded as a potential therapeutic target to inhibit T cell-mediated inflammation by restoring Treg function by blocking this signaling axis (58). Therefore, the timing and dose of OX40/OX40L should be precisely regulated to achieve the best anti-tumor effect.

### 4.3 Regulates the action of immune cells against tumors

**Figure 2:**
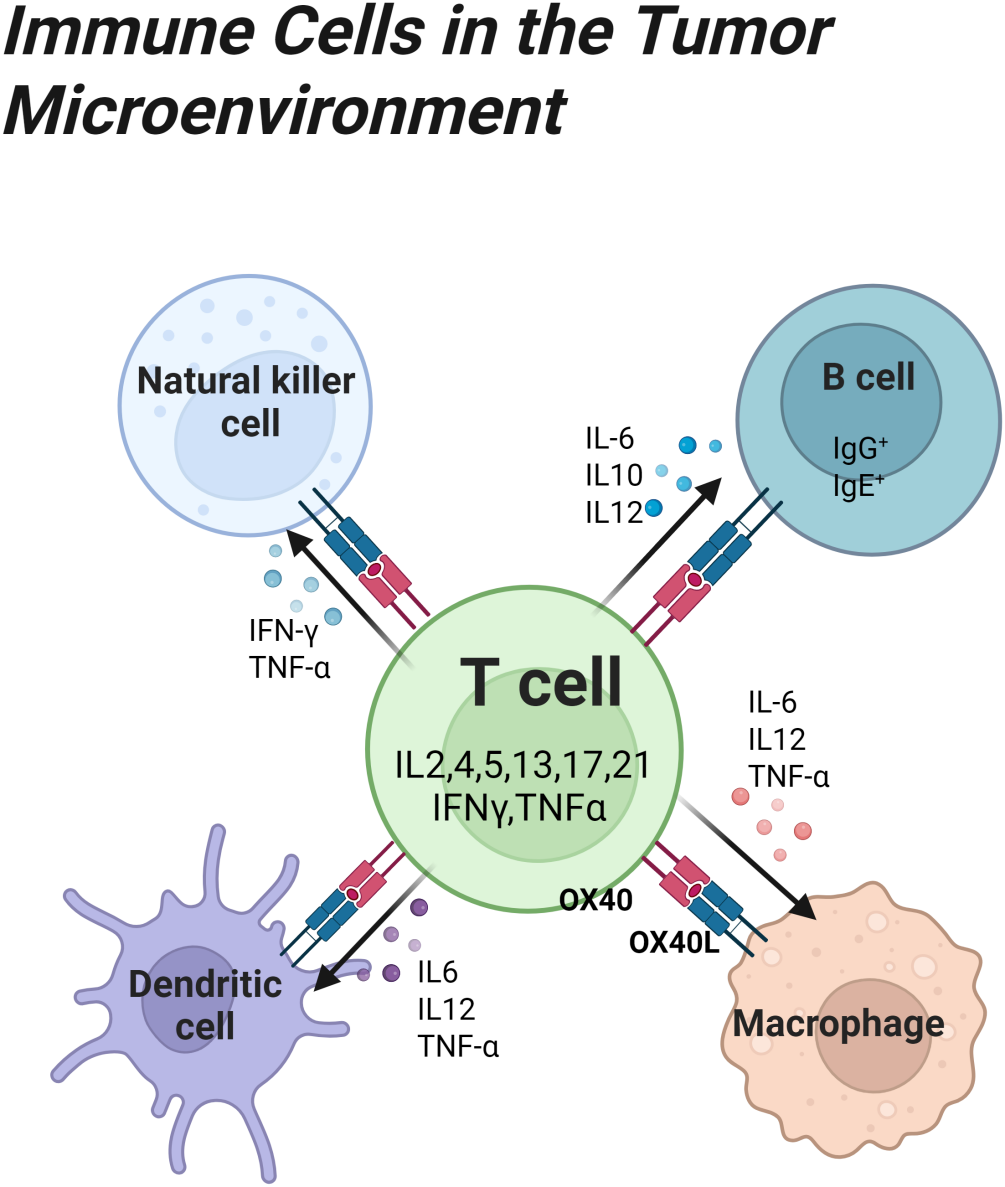
OX40/OX40L binding initiates a robust immune positive feedback loop. It promotes T cell subsets to secrete their characteristic cytokines IFN-γ, IL-4, IL-17 and so on, thereby specifically enhancing cellular immunity, humoral immunity and inflammatory response. At the same time, it transmits a signal to OX40L-expressing ligand cells to promote their secretion of key cytokines, thereby enhancing antigen presentation, antibody production and proinflammatory functions.

The OX40 signaling axis forms a bidirectional regulation between T cells and professional antigen-presenting cells. Activation of OX40 signaling can promote the activation and proliferation of professional presenting cells, causing them to secrete more cytokines and enhance the anti-tumor activity of the cells. **Figure 2** represents the bidirectional regulation between OX40 receptor T cells and OX40 ligand professional presenting cells.

#### 4.3.1 B cells

B cells engage T cells via OX40L to orchestrate follicular helper T cell (Tfh) differentiation, thereby supporting germinal center reactions and high-affinity antibody generation (23, 59). In the unique microenvironment of B cell malignancies such as follicular lymphoma, the OX40 axis undergoes functional inversion; by attenuating Tfh-mediated support to neoplastic B cells, it indirectly restrains tumor growth (60). OX40 signaling pathway can reshape the tumor-infiltrating B cell subsets, especially the regulatory B cells with immunomodulatory functions. These regulatory B cells often play a tumor-promoting role (61), yet OX40 activation skews B cell differentiation, selectively reducing intra-tumoral Breg frequency and relieving immunosuppression while potentiating antitumor humoral immunity (62, 63). Beyond this, the axis markedly enhances B cell activation and plasma cell transition, augments tumor-specific antibody production (64), upregulates the costimulatory molecule CD40 to strengthen B cell–T cell crosstalk, and amplifies T cell activation and expansion. Additionally, it tunes the metabolic state of B cells toward an antitumor phenotype (65).

#### 4.3.2 Dendritic cells

DCs are indispensable for initiating and sustaining antitumor immunity and constitute a principal cellular reservoir of OX40L.By enhancing antigen presentation and T cell priming, OX40L-expressing DCs engage naïve T cells within lymph nodes and deliver potent costimulatory signals that drive antitumor responses (66). Within the TME, metabolic perturbations such as hypoxia, lactate accumulation, and immunosuppressive cues frequently impair DC antigen presentation (67). The OX40/OX40L axis restores DC functionality and re-establishes their capacity to activate CD8+ T cells through multifaceted mechanisms (68). OX40 signaling activates the NF-κB pathway in DCs, partially reversing metabolic suppression and promoting proinflammatory cytokine secretion (69). Additionally, this signaling cascade fosters DC maturation and migratory competence, facilitating efficient capture and presentation of tumor antigens (70, 71). OX40 engagement further upregulates costimulatory molecules on DCs, thereby intensifying DC–T cell interactions and amplifying T cell activation and proliferation (21). Batf3-derived dendritic cells leverage OX40L together with ancillary cues to amplify antitumor T cell activity under PD-1/PD-L1 checkpoint inhibition, rendering them essential for optimal therapeutic efficacy (72).

#### 4.3.3 Natural killer cells

As an essential constituent of innate immunity, NK cells exert a critical antineoplastic function. Studies have shown that activation of OX40 signaling promotes NK cell activation and proliferation, thereby enhancing the release of IFN-γ and TNF-α, key proinflammatory mediators (73). The upregulation of activation receptors on NK cells augments their tumoricidal function, a process augmented by this signaling cascade (74). Furthermore, OX40 signaling improves NK cell survival within the tumor microenvironment by modulating their metabolic state (75). In the experiment, the combination of an OX40 agonist with chemotherapeutic agents induces a marked increase in intratumoral NK cell density, augments their IFN-γ production, enhances NK cell antitumor activity, and significantly suppresses tumor growth (76, 77).

#### 4.3.4 Tumor-associated macrophages

Within neoplastic tissues, tumor-associated macrophages (TAMs) exhibit pronounced phenotypic plasticity, and the OX40/OX40L signaling axis markedly influences their polarization state and functional properties (78). Studies indicate that this pathway can direct TAMs toward either the proinflammatory M1 or the anti-inflammatory M2 phenotype in response to microenvironmental cues, and that manipulating TAM polarization through this axis enhances their antitumor activity (79). Upon OX40 activation, TAMs are skewed toward the M1 phenotype, resulting in increased secretion of the proinflammatory cytokines IL-12 and TNF-α while suppressing M2-associated immunosuppressive functions. Concurrently, OX40 signaling augments TAM antigen uptake and presentation, indirectly priming the adaptive immunity. Upregulation of costimulatory molecules in tumor-associated macrophages through this pathway can enhance their interaction with T cells (80, 81). This highlights the essential function of the OX40/OX40L pathway in reprogramming immunosuppressive macrophages in the tumor microenvironment (82, 83).

## 5 Targeted therapy strategies

### 5.1 Agonist therapy

OX40 agonists are immunostimulatory monoclonal antibodies or derivatives that target OX40 and belong to the TNFR family. By binding to OX40 on T cells, these agonists mimic or potentiate the signal delivered by the natural ligand OX40L, thereby amplifying antitumor immunity (84). Stimulation of OX40 with agonist antibodies initiates NF-κB signaling via tumor necrosis factor receptor–associated factors. This activation potentiates T cell expansion, longevity, and effector functions, including cytokine secretion and a Th1-polarized response marked by IFN-γ production (37). During antitumor immune responses, OX40 stimulation augments TCR signal duration. This effect promotes the enrichment of CD8+ T cells within tumors along with the clonal expansion of high-affinity, tumor antigen-specific populations (85). Notably, OX40 agonists do not directly abrogate regulatory T cell suppression; rather, they indirectly expand Treg and conventional T cell (Tconv) populations by enhancing IL-2 production from Tconv cells (86). Thus, the core mechanism is driven primarily by the stimulation and clonal proliferation of T cells, not by the direct suppression of immunosuppressive functions. OX40 agonistic antibodies are currently available as bivalent antibody, Fab/scFv fragments, Fc fusion proteins, multivalent/bispecific constructs, and multimers. Different structural forms and their features have different functional advantages. Bivalent antibodies are engineered immunoglobulin molecules with two identical antigen-binding sites that specifically recognize the extracellular domain of the OX40 receptor. These usually consist of two identical heavy chain variable regions and light chain variable regions, forming two perfectly consistent Fab fragments, and bivalent antibodies are used clinically mainly for tumor immunotherapy (87). The Fab/scFv fragment targets OX40 Cystein-rich domain 2 by phage display technology, which has the advantages of humanization and avoids immunogenicity. Its bivalent structure can mimic the natural ligand to activate the T cell pathway, which is suitable for the adjuvant treatment of non-immunogenic cancer (87, 88). Fc fusion protein prolongs half-life and retains OX40 binding specificity through Fc domain, which can effectively inhibit OX40/OX40L interaction and is suitable for the treatment of autoimmune diseases (58, 89). Polyvalent/bispecific antibody can enhance the activity of the agonist by dual epitope targeting, and its dual epitope combination can synergistically activate T cell expansion and Treg suppression, which is suitable for combined immunotherapy (90). In clinical studies, bivalent antibodies are the predominant form and represent intact antibodies rather than fragments, fusion proteins, or multivalent constructs (91, 92). Traditional bivalent agonist antibodies cannot effectively induce the receptor to form the required trimer structure, which makes it difficult to achieve sufficient downstream signaling. Some researchers have compared the agonizing efficacy of bivalent antibody with that of the hexavalent CD40 agonist HERA-CD40L, and found that the high-valent polymeric structure can significantly enhance receptor clustering and signaling activation, thereby enhancing the function of antigen-presenting cells and promoting anti-tumor immune responses (93). Based on this idea, a novel hexavalent OX40 agonist promotes efficient receptor clustering through FC-independent cross-linking, showing stronger downstream signaling and T cell activation than traditional bivalent antibodies (94). These findings highlight the therapeutic potential of multivalent antibodies, which may provide a new direction for the development of TNFR family agonist antibodies. Clinically, these agents are principally investigated for cancer immunotherapy; nevertheless, studies have also demonstrated their efficacy against cutaneous inflammation and leukemia (95, 96). Multiple major pharmaceutical companies are developing OX40 agonists, most of which have entered clinical trials (**Table 1**).

**Table 1:** Examples of ongoing clinical trials targeting monoclonal antibodies against OX40.

Clinical ID	Format	Phase status	Name	Tumor type	Information by
NCT01862900	IgG1	I/II	MEDI6469	Metastatic breast cancer	Providence Health Services
NCT02274155	IgG1	I	MEDI0562	Head and neck cancer/Solid tumors/Ovarian cancer	Providence Health Services
NCT02315066	IgG2	I/II	PF-04518600	Metastatic carcinoma/malignancies	Pfizer
NCT02410512	IgG1	I	MOXR0916	Metastatic Solid tumors	Genentech
NCT02737475	IgG1	I/II	BMS-986178	Solid tumors	Bristol-Myers Squibb
NCT04387071	IgG4	I/II	INCAGN01949	Pancreatic cancer	University of Southern California
NCT03782467	mAb^2^	I	ATOR-1015	Advanced solid tumors	Alligator Bioscience AB
NCT04648202	mAb^2^	I/Ib	FS120	Advanced malignancy	invoX Pharma Limited
NCT04198766	sdAb_3_Fc	I	ES102/INBRX-106	Advanced solid tumors	Elpiscience Biopharma. Ltd
NCT03894618	PD-1-Fc-OX40L	I	SL-279252	Solid tumors	Shattuck Labs, Inc
NCT04215978	IgG1	I/II	BGB-A445	Advanced solid tumors	BeiGene
NCT02274155	IgG2	I/II	9B12	Head and neck cancer	Providence Health Services

### 5.2 Genetic engineering and cell therapy

**Figure 3:**
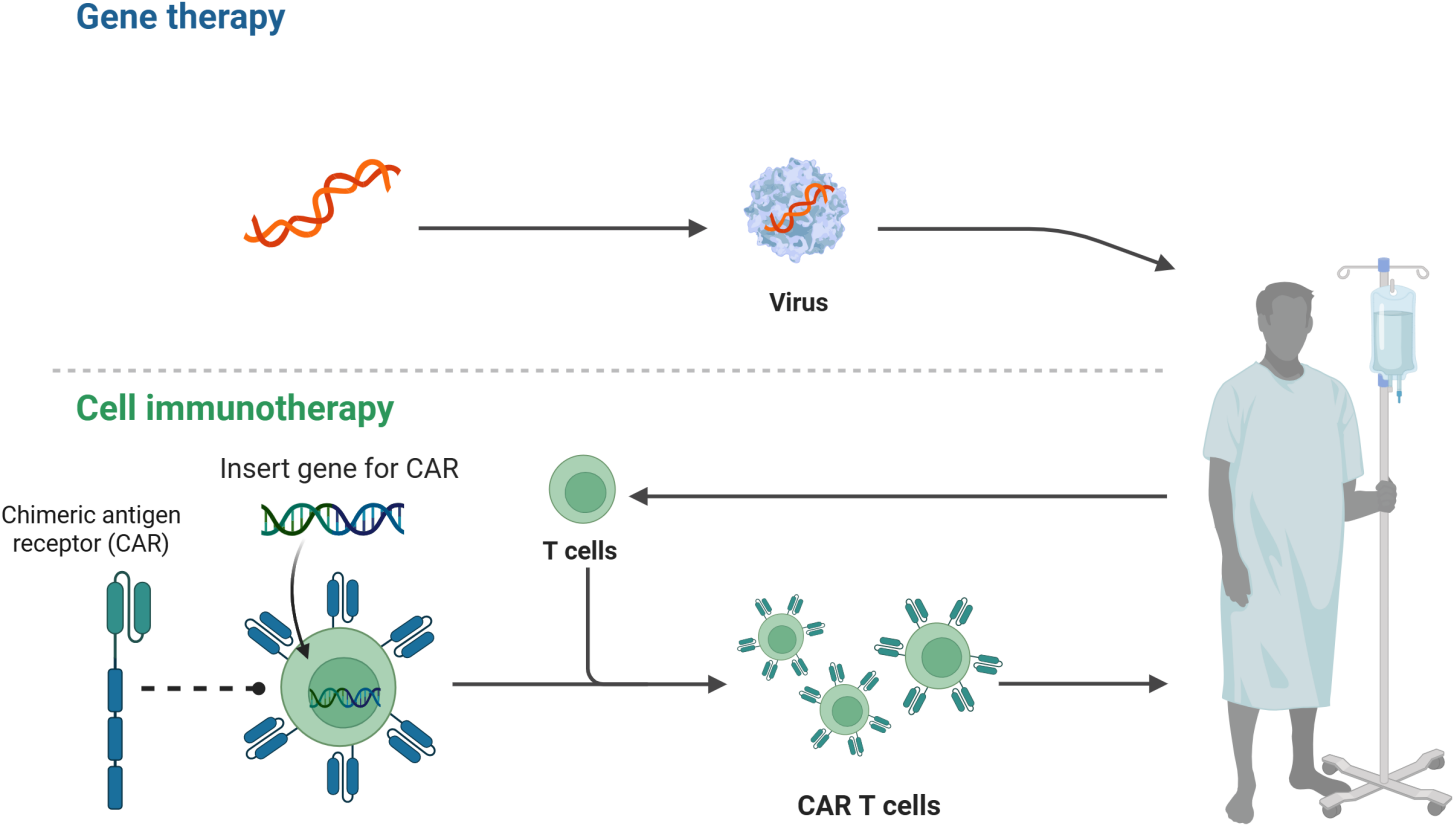
Principles of gene therapy and CAR-T cell therapy. The T cells were extracted from the patient and inserted with a CAR gene and a chimeric antigen receptor, which was finally infused back into the patient.

With the help of genetic engineering technology, OX40L expression can be precisely up-regulated in antigen-presenting cells or T cells, thereby enhancing the costimulatory signal of OX40/OX40L axis, optimizing the activity of T cell subsets, and further improving the anti-tumor efficacy of cell therapy. If combined with gene editing platforms, multi-target, programmable fine-tuning and safety control can also be achieved (97). Conceptually, OX40 binding could be enhanced by delivering the nucleic acid encoding OX40L to antigen-presenting cells or T cells engineered to overexpress OX40L with the use of a viral or nonviral vector (98, 99). Autocrine or paracrine OX40L signaling subsequently cooperates to activate T cells and augment antitumor activity (100). Targeted delivery systems can further restrict OX40L gene transfer to APCs or T cells within the tumor microenvironment, locally reinforcing the OX40/OX40L signal (101). The bidirectional regulatory capacity of OX40 signaling also permits precise control of T cell subset balance. OX40L overexpression expands CD4+ and CD8+ effector T cells, diminishes Treg suppressive function, and prolongs systemic T cell persistence by fostering memory T cell formation, thereby conferring long-term immunological protection (36, 102). Chimeric antigen receptor T cells are genetically engineered autologous or allogeneic T cell products that express synthetic receptors, endowing them with the capacity to recognize, activate, expand, and eradicate tumors (103). The specific interaction between OX40 and OX40L optimizes CAR-T cell functionality across multiple dimensions, including cell survival, functional differentiation, and TME modulation (104). Activation of the OX40/OX40L axis markedly enhances CAR-T cell survival and proliferation by triggering downstream NF-κB and PI3K-Akt signaling, suppressing pro-apoptotic proteins, and thus extending persistence and antitumor efficacy within the TME (105). In addition, OX40 costimulation can promote the transformation of CAR-T cells to an effector cell phenotype, and down-regulate the expression of exhaustion markers TIM-3 and PD-1, which effectively reverses the dysfunction of T cells and improves the killing ability of solid tumors (106). By reprogramming transcription factors, the OX40/OX40L axis orchestrates the differentiation of CAR-T cells into central memory T cells or stem cell–like memory T cells populations. This process fosters long-lasting antitumor immunity, enhances their sustained persistence *in vivo (*107). OX40 signaling also ameliorates the immunosuppressive TME via a dual mechanism: it inhibits the production of IL-10 and transforming growth factor-β by Tregs. Concurrently, it potentiates the expression of IFN-γ and IL-2 in the CAR-T cells. This altered cytokine milieu culminates in the engagement of innate immune cells; key populations including macrophages and dendritic cells are recruited and activated to mount a synergistic attack against tumors (57, 108, 109). Evidence further indicates that OX40 signaling enhances CAR-T cell vascular penetration and tumor infiltration efficiency (106). The cooperative action of OX40L therefore offers additional solutions to overcome limitations in CAR-T cell therapy, including insufficient persistence, TME-mediated suppression, and toxicity risks; nevertheless, optimal activation strategies and dosing regimens require further investigation. **Figure 3** illustrates the rationale for gene therapy and CAR-T cell therapies.

### 5.3 Combination treatment strategies

#### 5.3.1 Combined with immune checkpoint inhibitors

Although the OX40/OX40L signaling axis potentiates antitumor immunity, its efficacy is modulated by the complex tumor microenvironment. Tumor cells can secrete specific factors or alter metabolic states to impair OX40 signal transduction (109, 110). This has led to the development of combination therapies as a novel approach in cancer immunotherapy (111). The inhibitors of PD-1 and CTLA-4 have been widely used in clinical practice and have achieved encouraging results; concurrent or sequential administration with OX40 agonists is expected to yield superior therapeutic efficacy (112, 113). Mechanistically, PD-1/PD-L1 blockade releases T cells from inhibitory signaling, yet multiple suppressive factors persist in some TMEs. The addition of OX40 agonists delivers a positive costimulatory signal, thereby achieving a dual effect of stimulation and inhibition (42). The PD-1 immune checkpoint attenuates T cell activation via the suppression of TCR-induced phosphotyrosine signaling, whereas OX40 costimulation induces PI3K/AKT/mTOR activation without CD28 involvement, thereby rescuing T cell proliferative potential. A synergistic blockade of OX40 and PD-1 potently enhances CD8+ T cell functionality, marked by increased secretion of Granzyme B and IFN-γ while concurrently depleting Tregs in the tumor, thereby inducing significant immunomodulation (114).

#### 5.3.2 Combination with radiotherapy or chemotherapy

Radiotherapy produces synergistic antitumor activity with OX40 agonists through local tumor reduction and immunomodulation. Irradiation causes DNA damage that potentiates the presentation of tumor-associated antigens (TAAs) and damage-associated molecular patterns (DAMPs). As a result, DCs become activated and efficiently prime T cells (115). Concurrently, radiotherapy upregulates major histocompatibility complex class I and costimulatory molecules on tumor cells, thereby improving T cell recognition and cytotoxicity (116). OX40 agonists amplify the systemic antitumor immunity elicited by radiotherapy, potentiating the abscopal effect and suppressing the growth of distant metastases (117, 118).

Chemotherapeutic agents further synergize with OX40 agonists by inducing immunogenic cell death, which liberates TAAs and DAMPs (119). Clinical studies have shown that combining OX40 agonists with chemotherapy enhances antitumor responses, chemotherapy increases antigen availability and presentation, and OX40 signaling heightens T cell recognition of tumor antigens and reverses immune suppression within the tumor microenvironment (120). Chemotherapy also reduces the frequency of myeloid-derived suppressor cells (MDSCs) and Tregs, and co-administration with OX40 agonists further alleviates TME immunosuppression (121). Chemotherapy induces antigen release, radiotherapy enhances local immune activation, and OX40 agonists amplify systemic T cell responses. Chemotherapy and radiotherapy decrease Treg and MDSC populations, while OX40 agonists block Treg function and augment effector T cell activity.

## 6 Research progress

### 6.1 Preclinical studies

At present, gene therapy or CAR-T cell therapy with OX40/OX40L as the direct intervention target has not yet entered registered clinical trials. The most widely studied strategy for OX40L is OX40 agonist therapy.Preclinical studies demonstrate that OX40 agonists, whether used as monotherapy or in combination, elicit robust antitumor activity in murine tumor models. Ma and colleagues reported that pancreatic ductal adenocarcinoma appears to evade immunity by inducing suppressive T cells. Compared with controls, mice receiving antiOX40 monotherapy exhibited improved survival; Coadministration of antiOX40 and antiPD-1 led to Treg depletion concurrent with an expansion in CD4+ and CD8+ T cell populations, resulting in complete tumor eradication (48). In a bladder cancer model, an OX40 agonist antibody enhanced CpG-mediated antitumor activity and prolonged overall survival (122). In colon cancer models, Zhang et al. showed that the OX40 agonist SHR-1806 markedly increased IFN-γ secretion, promoted antitumor T cell responses, and preserved both antibody-dependent cellular cytotoxicity and complement-dependent cytotoxicity to eliminate Tregs and achieve tumor regression (123). In another colon cancer study, Jiang et al. employed the OX40 agonist antibody BGB-A445 and demonstrated that it activated T cells without compromising NK cell function, while antibody-dependent cellular cytotoxicity depleted Tregs *in vitro* and *in vivo*, resulting in superior immunostimulation and antitumor activity (22). In melanoma models, an OX40 agonist antibody enhanced CD8+ T cell cytotoxicity, fostered tumor-specific memory formation, delayed tumor progression, and extended survival (110). In a glioma study, combination therapy with an OX40 agonist elevated the proportion of Th1 CD4+ T lymphocytes, reversed intracerebral T cell exhaustion, decreased PD-1 expression, and augmented Th1-mediated antitumor immunity (124). Guo and colleagues conducted the first study to combine antiOX40 with antiPD-1 in an ovarian cancer model; the regimen expanded CD4+ and CD8+ T cells, reduced Treg and MDSC numbers, induced a local immunostimulatory milieu, and markedly inhibited tumor development (125). In mice with breast cancer, co-administration of Sm16 and antiOX40 elicited potent antitumor effects, attenuated tumor growth and metastasis, and reinforced resistance to rechallenge, accompanied by robust tumor-specific peripheral memory IFN-γ responses (126).

We found that some trials used agonist antibodies that retained Fc effector function. The potential to mediate the effects of ADCC is exploited to both excite effector T cells and eliminate regulatory T cells (89, 127). However, excessive Treg consumption increases the risk of immune-related adverse events. In order to reduce this situation, the agonist should be selected for the IgG2 or IgG4 indolent subtype with weak binding to FcγR, or the Fc silencing mutant (128–130). Collectively, these preclinical studies demonstrate the strong antitumor activity of OX40 agonist antibodies, providing a solid foundation for their clinical translation. The progress of related clinical investigations is presented in the following section.

### 6.2 Clinical trials of OX40 agonist monotherapy in tumor intervention

**Table 2** provides an overview of some clinical trials of OX40 agonist antibodies, both as monotherapy and in combination. The safety and efficacy of BAT6026(a humanized IgG1 monoclonal antibody)were assessed in a phase I study (NCT05105971) (131) involving individuals with advanced solid tumors. Escalating doses of 0.01 to 10 mg/kg were administered intravenously to 30 enrolled patients on day 1 of each 21-day cycle. During the expansion phase, a cohort of 6 participants was treated at a dose of 10 mg/kg. The antiOX40 agent demonstrated a favorable safety profile and was well tolerated. No dose-limiting toxicities were reported and the maximum tolerated dose was not attained in this trial. Regarding antitumor activity, neither complete nor partial responses were observed among participants. Stable disease (SD) was achieved in 10 subjects, yielding a disease control rate (DCR) of 38.5%.

**Table 2:** Example of OX40 agonist antibodies in clinical studies.

Name	Phase	Combination	Type of mouse tumor	Type of OX40 therapy	Effect
BAT6026	I	Single agent	Advanced cancer	Anti-OX40,0.01-10mg/kg, IV, Q3W.	N=30, ORR 0%, DCR 38.5%
MEDI6469	I/II	Single agent	HNSCC	Anti-OX40,0.4mg/kg, IV, day1.3.5	N=17, OS 82%; PFS 71% at 3 years
MEDI0562	I	Single agent	Advanced Solid tumors	Anti-OX40,0.03-10mg/kg, IV, Q2W.	N=55, ORR 4%; PR1, DCR 22%
INCAGN01949	I/II	Single agent	Advanced Solid tumors	Anti-OX40,7-1400mg, IV, Q2W.	N=87, ORR 1.5%; PR1
MOXR0916	I	Single agent	Advanced Solid tumors	Anti-OX40,0.2-1200mg, IV, Q3W.	N=172, ORR 1.2% DCR66%
BGB-A445	Ib	Tislelizumab	Advanced Solid tumors	Anti-OX40,0.03-40mg/kg, IV, Q3W. Anti-PD-1,200mg, IV, Q3W.	N=30, ORR 23%; PR 7
GSK3174998	I	Pembrolizumab	Advanced Solid tumors	Anti-OX40,0.03-10mg/kg, IV, Q3W. Anti-PD-1,200mg, IV, Q3W.	N=138, Single agent ORR0%. Combination with aPD-1, ORR 8%; CR 2; PR 4
PF-04518600	I/II	Utomilumab	Advanced Solid tumors	Anti-OX40,0.1-3mg/kg, IV, Q2W. Anti-4-1BB,20/100mg, IV, Q4W.	N=57, ORR 3.5%; PR 2; DCR 35%
BMS986178	I	SD-101 Radiation	LG-B-NHL	Anti-OX40,0.3mg/kg, IV, Day8.15.22 SD-101,1-8mg, IT, Day8.15.22.29.36 RT, Day1.	N=29, ORR 31%; CR 4; PR 5
BMS986178	I/II	Nivolumab Ipilimumab	Advanced Solid tumors	Anti-OX40,20-320mg, IV, Q2W Anti-PD-1,240-480mg, IV, Q2W Anti-CTLA-4,1-3mg/kg, IV, Q3W	N=165, Single agent ORR 0%(n=20), Combination therapy ORR 0-13%(n=145)
PF-04518600	I/II	Avelumab UtomilumabRadiation	Advanced gynecologic malignancies	Anti-OX40,0.3mg/kg, IV, Q2W Anti-PD-L1,10mg/kg, IV, Q2W Anti-4-1BB,20/100mg, IV, Q4W	N=35,Avelumab+Utomilumab: ORR11%;PR1(n=9),Avelumab+Utomilumab+PF04518600:ORR2.9%,DCR37.1%,(n=35)

CR, complete response; DFS, disease-free survival; ORR, overall response rate; OS, overall survival; PR, partial response; PFS, Progression Free Survival; HNSCC, Head and Neck Squamous Cell Carcinoma; LG-B-NHL, Low-Grade B-cell Non-Hodgkin Lymphoma.

To evaluate the safety and feasibility of neoadjuvant antiOX40 therapy, patients diagnosed with head and neck squamous cell carcinoma (HNSCC) received preoperative administration of an OX40 agonist antibody MEDI6469(a humanized IgG2a monoclonal antibody) in a clinical trial (NCT02274155) (132). A total of 17 participants were treated with the antiOX40 agent at a dosage of 0.4 mg/kg on days 1, 3, and 5. The therapy exhibited a favorable safety profile and was well tolerated, with only grade 1 or 2 adverse events observed. After a median follow-up of 39 months, the overall survival (OS) and disease-free survival (DFS) rates were 82% and 71%, respectively. Immunological profiling indicated an elevation in CD4+ and CD8+ T cell counts after treatment. Furthermore, expansion of CD103+CD39+CD8+ tumor-infiltrating lymphocytes was noted in a subset of patients. This increase was correlated with restrained tumor progression and did not elevate the risk of postponing surgery.

The safety and antitumor activity of the OX40 agonist antibody MEDI0562(a humanized IgG1 monoclonal antibody) administered as a single agent were investigated in a phase I/II study (NCT02318394) (133) involving patients with advanced solid malignancies. The participants were administered intravenous infusions of the antibody biweekly, at dose levels escalating from 0.01 to 10 mg/kg. The therapy demonstrated a manageable safety profile and was well-tolerated. Among 55 evaluable subjects, an objective response rate (ORR) of 4% was observed, including two partial responses. Immunological analyses revealed a marked elevation in Ki67-positive CD4+ and CD8+ memory T cell populations after treatment. This immunostimulatory effect was concomitant with a decrease in OX40+Foxp3+ Tregs in the tumor microenvironment.

In patients presenting with advanced solid malignancies, the therapeutic efficacy of INCAGN01949(a human IgG4 monoclonal antibody) designed to activate OX40, was assessed in a phase I/II clinical study (NCT02923349) (9). A dose-escalation study of the antibody as monotherapy was conducted over 14-day cycles, with doses ranging from 7 to 1400 mg. Among the 87 patients, the ORR was 1.5%, with one patient achieving a partial response. Twenty-three patients attained SD, with one patient maintaining SD for over six months. No safety concerns were identified with monotherapy; however, the treatment did not enhance T-cell proliferation or reduce the number of Tregs.

The safety, tolerability, and antitumor efficacy of the OX40 agonist antibody MOXR0916(a humanized IgG1 monoclonal antibody) were assessed in a phase I clinical trial (NCT02219724) (134) involving individuals with advanced solid malignancies. The participants were administered the antibody intravenously in a three-week cycle, with doses escalating from 0.2 to 1200 mg. A favorable safety and tolerability profile was observed, wherein most treatment-emergent adverse events were grade 1 or 2. Among the 172 treated participants, the ORR was 1.2%, which consisted of two partial responses. Immunological analyses demonstrated an elevation in CD8+ T cell numbers and increased cytokine concentrations in a subgroup of participants.

Despite limited single-agent efficacy, OX40 agonists have been shown to be well tolerated. Considering the complexity of the tumor microenvironment and the activation efficiency of agonists themselves, the combination of other treatment options still has clinical development prospects.

### 6.3 Combination therapy trials

Preliminary data from a phase I/II clinical study (NCT04215978) (135) involving subjects with advanced solid malignancies were disclosed at the annual conference of American Society of Clinical Oncology. The investigation assessed the safety and antitumor activity of BGB A445(a humanized IgG1 monoclonal antibody), administered both as a single-agent therapy and in combination with an anti PD 1 antibody. The data revealed that no severe adverse events occurred in the enrolled participants. Therapeutic interventions were generally well tolerated, and no dose limiting toxicities were detected. Among the 50 patients receiving monotherapy, the ORR was 4%, with two partial responses. In the combination therapy cohort, 30 patients received BGB-A445 plus 200 mg Tislelizumab (a humanized antiPD-1 IgG4 monoclonal antibody), resulting in an ORR of 23% with seven partial responses.

The clinical efficacy of the OX40 agonist antibody GSK3174998(a humanized IgG1 monoclonal antibody) was examined in a phase I study (NCT02528357) (136) involving patients with advanced solid tumors, evaluating both single-agent and combination regimens with immune checkpoint blocking agents. NO clear dose-toxicity relationship was identified, and the study did not reach the maximum tolerated dose. Adverse effects were predominantly grade 1 or 2 in severity. In Part 1 (monotherapy), which included 45 patients, the ORR was 0% and the DCR was 9%. In Part 2 (combination therapy), 96 patients received GSK3174998 in combination with 200 mg pembrolizumab (a humanized antiPD-1 IgG4 monoclonal antibody), achieving an ORR of 8%, which included two complete responses and four partial responses.

A phase I/II clinical study (NCT02737475) (137) evaluated the OX40 agonist antibody BMS986178(a fully human monoclonal antibody) as a monotherapy and in combination with other agents in patients with advanced solid tumors. The maximum tolerated dose was not achieved. The treatment demonstrated a favorable safety profile; most adverse events were grade 1 or 2 in severity, and grade 3 or 4 events occurred in a minority of patients. The ORR was 0% for monotherapy and ranged from 0% to 13% across combination therapy cohorts.

The safety and efficacy of a combination regimen containing the OX40 agonist antibody PF-04518600(a humanized IgG2 monoclonal antibody), nivolumab(a fully human antiPD-1 IgG4 monoclonal antibody), and ipilimumab (a human antiCTLA-4 IgG1 monoclonal antibody) were evaluated in a phase I trial (NCT02315600) (138) involving patients with advanced solid tumors. The trial employed a dose escalation design. The patient tolerated the combination therapy well. Grade 3–4 adverse events occurred in 28 of the 57 assessable patients. Partial responses were achieved in two melanoma patients, and stable disease was documented in 18 patients. A DCR of 35.1% was observed across all the dose cohorts.

A phase I study (NCT02317747) (139) assessed a triple therapy regimen comprising the OX40 agonist PF-04518600 in combination with avelumab (a fully human antiPD-L1 IgG1 monoclonal antibody) and utomilumab(a fully human anti4-1BB agonist IgG2 monoclonal antibody) in individuals with gynecologic cancers. This combination therapy resulted in a favorable safety profile. Only grade 1 or 2 adverse events were documented, with no dose-limiting toxicities, and the regimen was generally well tolerated. DCR was 78% in patients receiving avelumab and utomilumab alone. In a group of nine subjects administered avelumab plus utomilumab, an ORR of 11% was achieved, including one partial response. Among the 35 participants treated with the triple antibody combination, the ORR was 2.9% with a DCR of 37.1%.

Combination therapy is superior to monotherapy, but the optimal combination regimen and dose have not yet been determined, which also increases the frequency of immune-related adverse events in patients, and there are also many problems and challenges in the treatment. More sample studies are needed to explore the value of OX40 agonist combination therapy in the future.

### 6.4 Terminated or failed studies

Despite the remarkable efficacy of OX40 agonists in animal models, a number of clinical trials have been terminated early due to limited efficacy and intolerable immune-related adverse events. The agonist PF-04518600 showed shortcomings in a phase I trial (NCT02315066). Due to the lack of cross-linking with FcγR, the ORR of monotherapy was only 5.8% (n=142), while peripheral T cell activation signals were still not seen after increasing the dose, and grade 3 or above hepatotoxicity appeared in advance. The experiment was terminated early (140). Almost at the same time, another FC-enhancing antibody MOXR0916 in (NCT02410512) resulted in an ORR of 4% (n=51) due to the low proportion of intratumoral OX40^+^ T cells, and the trial was stopped in November 2019 due to the grade 3 rash/diarrhea blocking dose escalation (141). In another phase I trial (NCT02528357), GSK3174998 was used in combination with pembrolizumab in patients with advanced cancer, but development was stopped due to insufficient efficacy (136). One agonist, MEDI0562, failed in a trial of advanced solid tumors (NCT02705482) because it did not show any objective efficacy, with a grade ≥3 immune-related adverse event rate of 22% (n = 58) (142). INCAGN01949 with FcγR enhanced design was terminated in 2022 in (NCT04387071) due to weak peripheral and intratumoral pharmacodynamic signals, ORR of 1.15% (n=87), and 9% grade 3 rash (143).

The reasons for the failure of the earlier OX40 agonist clinical trials, which were terminated, were analyzed. The agonist failed to elicit an effective pharmacodynamic response *in vivo*, as indicated by insufficient intratumoral OX40^+^ T cell infiltration, and no available pharmacodynamic biomarkers were found in peripheral blood to monitor its activity. Patients with tumors with high OX40 expression were not screened, which limits the efficacy of the agonist in the target population. On the safety front, the high dose resulted in frequent immune-related adverse events. In addition, when combined with other immunosuppressive agents, the treatment regimen does not show better synergy, but instead aggravates the safety problem due to the superimposed toxicity. Taken together, these trials were stopped because of limited clinical benefit. This is also a challenge in the development of OX40 agonists.

## 7 Safety

Although OX40 agonist antibodies have shown strong anti-tumor potential in preclinical studies, the overactivated immune system may also injure normal tissues and organs while attacking tumors. Therefore, immune-related adverse events (irAEs) are an important concern in immunotherapy. Immune-related adverse events were graded in clinical trials according to the Common Terminology Criteria for Adverse Events, version 5.0.

### 7.1 Potential mechanisms

OX40 signaling further enhanced the function and migration of activated CD4^+^ and CD8^+^ T cells in the presence of tumor antigens, leading to low-level antigen cross-recognition and inflammation in normal tissues such as intestine, skin and liver (144–147). Studies have shown that OX40 agonists can weaken the suppressive phenotype of Tregs and promote their clearance, thereby breaking local immune tolerance and exacerbating autoimmune responses (84). At the same time, intense T cell activation can be accompanied by the increase of IFN-γ, IL-2, TNF-α and other inflammatory factors, which increase the probability of triggering systemic inflammatory response (26, 148).

### 7.2 Safety data from clinical studies

So far, most OX40 agonists are still in the early stage of clinical research, and their safety databases are still being accumulated. Several experimental studies have shown that OX40 agonists have a relatively good safety profile when used as monotherapy. A phase I clinical trial (NCT05105971) in patients with advanced cancer (n=30) showed that OX40 agonist BAT6026 0.01-10mg/kg monotherapy had a good safety profile, and immune-related adverse events were grade 1-2, distributed in different dose groups, mainly rash (131). In another trial evaluating the efficacy of agonist antibody MEDI6469 0.1-0.4mg/kg in HNSCC (NCT02274155) n=20, adverse reactions were mainly rash and diarrhea, all of which were grade 1-2, and no grade 3 or above irAEs were found (132). In the (NCT02318394) n=57 trial, which evaluated the efficacy of single-agent MEDI0562 at 0.03 to 10mg/kg in the treatment of advanced solid tumors, 26.3% (15/57) of patients developed grade 1–2 irAEs. One grade 3 rash irAEs occurred in the 10mg/kg dose group (1.8%). In the second part of the trial (n=15), MEDI0562 3mg/kg was given in combination with Atezolizumab 1200mg. Grade 1–2 adverse events, mainly rash, occurred in 67% (10/15) of patients, grade 3 adverse events occurred in 13% (2/15) of patients. In this trial, patients with grade 3 adverse effects were permanently discontinued, treated with glucocorticoids, and recovered (133). In another experiment of GSK3174998 in the treatment of solid tumor patients (NCT02528357), 20% (9/45) of the patients in the monotherapy group received 0.03-10mg/kg had grade 1–2 irAEs, mainly characterized by rash and mild diarrhea. After combined treatment with pembrolizamab 200mg, 32.3% (31/96) patients developed grade 1–2 irAEs and 10.4% (10/96) developed grade 3 irAEs. Patients with grade 3 irAEs were permanently discontinued and treated with glucocorticoids, and no further experiments were performed (136).

In conclusion, the incidence and severity of irAEs were higher in the combination group compared with the monotherapy group. Although the efficacy of combination therapy is better, it also increases the frequency of irAEs. This makes clinical trials of this combination strategy a great challenge, which also requires investigators to weigh its efficacy benefits against potential risks. Looking forward, the key to overcome the irAEs challenge is to develop predictive biomarkers and optimize the dosing regimen, in order to maximize the efficacy while minimizing the potential risks to the body.

## 8 Discussion

Although OX40/OX40L targeted therapy has shown great potential in preclinical experiments. However, clinical trials have not shown the same efficacy. Moreover, some clinical trials have been stopped due to low efficacy and serious side effects. To date, no OX40 agonist clinical trials have successfully progressed beyond phase II. And there are many problems and research gaps in clinical trials. The efficacy of OX40 agonist monotherapy is limited and the objective response rate is low. Although the combination of OX40 agonist and other immune checkpoint inhibitors improves the response rate, it also increases the incidence of irAEs in patients, showing no obvious advantage. In addition, inadequate activation of receptor signaling can affect its efficacy. The optimized downstream signal of the OX40 signaling pathway requires multivalent activation, while the traditional bivalent OX40 agonist cannot effectively induce receptor clustering, and the effect of activating T cells is not good, so it cannot achieve the expected antitumor effect. In the future, it is necessary to develop new, more efficient agonist molecular structures that can activate signaling pathways at lower doses. There is a mismatch between biological activity and clinical benefit, with receptor and T-cell activation signals detected in the peripheral blood and tumors in multiple trials, but without inhibition of tumor growth or survival benefit. This is because OX40 expression is upregulated approximately 3 days after the full activation of T cells. However, many trials use biweekly or triweekly dosing regimens, which may miss the optimal window for signal costimulation. To this end, we can match the time window of T cell activation by precise dosing times rather than fixed long intervals. OX40 agonists have had limited clinical benefits as monotherapy, but combining them with other immune stimuli, such as radiotherapy and chemotherapy, can create a larger activation window and optimize drug delivery. In addition, the drug escalation strategy was used inappropriately, and the dosing schedule did not match the immunokinetic. In clinical trials, researchers tend to push agonists to the maximum tolerated dose. After signal activation, T cells expand rapidly and easily enter the activation exhaustion state, which reduces the therapeutic potential. We should avoid sustained high-dose therapy in favor of immunodynamics-based dose exploration to identify the lowest effective dose that activates T cells while avoiding exhaustion. The optimal combination of OX40 agonists with other therapies has not been established, and although they are often combined with immune checkpoint inhibitors or other agents in clinical trials, these regimens have not been optimized for the biological mechanism of OX40 and may have poor synergistic effects and the risk of additive toxicity. The optimal treatment combination can be designed according to the mechanism of action, and the timing and sequence of administration can be optimized to achieve the best efficacy while reducing the risk of immune-related adverse events.

There are differences between patients; most of the patients participating in phase II clinical trials have advanced solid tumors, patients are not screened, and only a few patients may respond to treatment. No study to date has screened patients with high OX40 expression for OX40 agonist therapy; therefore, this study could be performed to maximize efficacy. Clinical trials have shown that OX40 agonists combined with chemoradiotherapy have preliminary efficacy, but there are no studies on the combination of the three, which may lead to better efficacy. In addition, there is a lack of specific biomarkers that can predict the efficacy of OX40 agonists and the risk of irAEs. In the future, there is an urgent need to develop such biomarkers to achieve precise clinical application of this strategy.

Multiple trials and reports have shown that compared with traditional immunotherapy, monotherapy with PD-1/PD-L1 and CTLA-4 inhibitors has relatively higher objective response rates than monotherapy with OX40 agonists. However, the incidence of immune-related adverse events is lower than that of traditional immunotherapy (149–154). This suggests that the advantage of current OX40 agonists is tolerability rather than efficacy and that more efficient agonists need to be developed in the future.

## 9 Conclusion

The OX40 agonist antibody is used to optimize the host’s anti-tumor immune response from multiple aspects and inhibit tumor growth. Although it has a wide safety range, its clinical benefits are limited. When combined with other immunosuppressants, it has not achieved the expected effect. Most trials have not reached the maximum tolerated dose, but various degrees of adverse reactions have been reported. Therefore, further research is needed to expand the sample size, adjust the dosing regimen, and determine its value in clinical practice. In the future, OX40 agonist therapy should shift from simple immune activation to precise, safe and efficient activation. Molecular design is moving towards multi-specificity, and the administration strategy will shift from systemic intravenous administration to more effective targeted delivery systems, aiming to limit the co-stimulatory signals in the tumor microenvironment while minimizing systemic toxicity. However, for the time being, it may be more suitable as an adjunct to traditional immunotherapy.
